# Access into professional degrees by students with disabilities in South African higher learning: A decolonial perspective

**DOI:** 10.4102/ajod.v8i0.514

**Published:** 2019-06-10

**Authors:** Sibonokuhle Ndlovu

**Affiliations:** 1 Ali Mazrui Centre for Higher Education Studies, University of Johannesburg, Johannesburg, South Africa

**Keywords:** students with disabilities, higher learning, access, decolonial theory, specific impairments, professional degrees

## Abstract

**Background:**

Former historically disadvantaged social groups such as women, black people and those with disabilities are expected to participate in the skilled labour force that South Africa has pledged to produce for the 21st century. However, in the South African context, research widely neglects access of those into professional degrees in higher learning. There is a need for such an exploration because people with disabilities have been found to be excluded from professional employment.

**Objectives:**

Using decolonial theory, this empirical study sought to explore obstacles confronted by students with disabilities at entry in a specific institution of higher learning in South Africa. The aim was to unveil the invisible obstacles and their causes for an effective intervention.

**Method:**

A qualitative research design was adopted and in-depth interviews were conducted to collect data from the participants. This particular dimension of research method was chosen to enable dialogue and development of partnership, which is important for collecting rich data.

**Results:**

While policies of inclusion still enabled access of all students into professional degrees, there were however inequitable practices, alienation and inequality that excluded students with disabilities at entry. Obstacles seen at surface level were not the real ones; the real ones were the deep-seated issues of coloniality.

**Conclusion:**

If the underlying causes of obstacles at entry are not visible to students with disabilities themselves and the responsible stakeholders, students might continue to be oppressed on entry into the professional degrees and in higher learning generally. Obstacles can only be dismantled when there is an awareness about their deep-seated causes.

## Introduction

Access of students with disabilities into higher learning has shown a general increase globally. The United Kingdom, for example, has well-developed policies of inclusion (Fotim Project Report 2011) (RB, 2a), which enhance access to higher education for students with disabilities, through established support, services, processes and structures (Chataika 2007). In Turkey, the limitations are imposed by poor resources (Arsian-Ari & Inan 2010). For example, students with disabilities in particular do not have access to assistive and Internet technologies (Ozel, Inan & Sezer 2004). In Canada, it is the structural barriers that are limiting, namely, while efforts are made for physical structures to be accessible, those with disabilities receive less attention and remain excluded (Mullin & Preyde 2013). The United States of America has good policies, but poor implementation presents barriers for the inclusion of students with disabilities in the curriculum (Mosia & Phasha 2017). In the African context, all students are expected to have access to higher learning, and their voices to be heard because of the philosophy of Ubuntu (Shanyanana & Waghid 2016) where everyone is ‘supposedly’ included. However, it has been revealed in a number of studies that in developing countries in Africa, access to higher learning is still limited. In Zimbabwe (Chataika 2007), Namibia (Hugo 2012) and Lesotho (Mosia & Phasha 2017) although students with disabilities access higher learning, they are excluded from curricula because of the lack of adequate funding, and structural and attitudinal barriers.

In the South African context, studies by Carrim and Wangenge-Ouma (2012), the Council of Higher Education (2013) and the information shared at the Second National Higher Education Summit (2015) reveal an increased access into higher education of diverse students. ‘Diverse’ in this context refers to different social groups of students, defined in terms of ethnicity, religion, race, gender, sexual orientation and disability, in both historically disadvantaged and advantaged institutions of higher education. Howell (2005) remarks that in basic education (i.e. in schools) the government has made remarkable improvements to enable diverse learners to gain access. However, although there has been increased access into higher education, students with disabilities still confront obstacles in accessing higher learning generally, and professional degrees specifically. The problems of access for students with disabilities have generally been found to start from schooling. This fact is mirrored through very few people with disabilities being engaged in professional employment (Ramutloa 2010), and obstacles at entry to professional degrees could be the reason why people with disabilities are typically found in non-professional employment (Swartz & Schneider 2006).

In this article, decolonial theory is used as a theoretical lens to explain the underlying causes of the obstacles confronted by students with disabilities when entering professional degrees. The articles seeks to make the invisible visible so that when obstacles are revealed from this perspective, along with their causes, an effective intervention aimed at the underlying causes can be planned. It further seeks to contribute to the issues of persons with disabilities also being afforded the opportunity to access professional degrees in higher education and, consequently, participate in the professional labour force that South Africa seeks to produce for the global market of the 21st century (Carrim & Wangenge-Ouma 2012).

As professional employment and participation in the professional labour force start from entry into the professional degrees, this article first outlines what is required to enter professional degrees in the South African higher learning context. It then expounds extensively on decolonial theory for its potential to illuminate that which might be invisible and to bring it to the fore (Dastile & Ndlovu-Gatsheni 2013). The main themes perceived by the participants as obstacles to enter the specific professional degrees are then reported, which are specifically access and alienation, equality and inequality, intersectionality and the tension of impairment and disability. Decolonial theory is then applied to discuss the specific themes, in the process making visible what could be actual obstacles and their underlying causes.

### Access to professional degrees in South Africa

Entry requirements for professional degrees vary from one degree programme to another. The variance is in terms of academic marks obtained at school, compulsory subject requirements and/or admission procedures. The requirements may also vary from one institution to another. Access to professional degrees for all students, including those with disabilities, is backed by policy. Broadly speaking, the present democratic South Africa has a constitution (Republic of South Africa [RSA] 1996) and legislation, such as the *Equality and Prevention of Unfair Discrimination Act* (RSA 2000) and the *Employment Equity Act* No. 55 of 1998 (RSA 1998), that prevent unfair discrimination and promote equity and equal opportunity and representation in terms of employment. It also has inclusive education policies, like the Education White Paper 6: Special Needs Education (EWP6) (Department of Education [DOE] 2001) and the Education White Paper 3: A Programme for the Transformation on Higher Education (DOE 1997). These policies were developed to enable all students to access basic education and higher learning in general, and to enter professional degrees and, consequently, professional employment. With such policies, I argue that without delving deep into the empirical exploration, it can be assumed that access into professional degrees might be without obstacles for all students, including those with disabilities.

This article focuses on entry into three specific professional degrees: Law, Medicine and Education. These degrees were chosen because the Department of Higher Education and Training reported an imbalance between the higher learning output of specific professions and national skills. Because of limited entry into specific professional degree programmes, there has been a shortage of these professional skills in the country (Department of Higher Education and Training [DHET] 2011). It is important therefore to focus on the particular degree programmes to understand whether diverse students, including those with disabilities, have access into them at entry. At the institution where the research was conducted, Medicine and Law degrees require higher marks in matriculation than an Education degree. Medicine specifically requires science subjects as compulsory for entry.

The institution of higher learning does not deny students with disabilities entry into Law, Medicine, Education or any other professional degree. Students with disabilities are merely advised on the difficulties they might experience in taking a particular programme, looking at the demand of the programme versus the impairment of the student and its severity. For example, severe communication disorders and dyslexia might hinder entry into Law. While all students confront obstacles at entry, there are additional ones specifically faced by students with disabilities because their needs are unique (Ndlovu & Walton 2016).

### Theoretical perspective

Several theories provide a lens which might have informed the focus of the current study. For example, scholars in the disability field often employ the framework provided by critical disability studies (CDS) to understand issues of disability and oppression of people with disabilities (Meekosha & Shuttleworth 2009; Tremain 2005). Furthermore, CDS includes a number of theoretical positions (Spagnuolo 2016), and it draws together works produced in different studies to better understand disability issues across the world (Shildrick 2012). It constructively critiques and problematises specific disability issues so as to generate new ways of understanding. One of the aims of CDS is to improve the living conditions of all diverse persons, including those with disabilities who are undervalued and discriminated (Meekosha & Shuttleworth 2009). The proponents of CDS comprise a range of post-conventionalists, post-structuralists and post-colonialists, who include, among others, Meekosha and Shuttleworth (2009), Shildrick (2012) and Goodley (2014). They critique, among other issues, ableism and disablism and seek to create a new understanding of disability in light of intra- and/or intersectionality, suppressed voices and acknowledgement of difference. Most striking is the work of post-colonialists like Grech (2015) and Soldatic (2015) who sought to shift the understanding of disability from a Eurocentric Global West perspective to include voices from the South. Understanding of disability from this perspective could result in decolonisation of disability.

The Social Model of Disability as a theoretical framework proposes that disability is a social construct. Gallagher, Connor and Ferri (2014) argue that the social model does not emphasise biological determinism, which reinforces that disability does not result from impairments but from a social construct. This results in some quadrants of society remaining out of reach for some people. When considering the importance of CDS and Social Model of disability as theories for a disability study, they could have been the ones informing this study. However, I chose to apply decolonial theory because it not only explains oppression but goes further to expose the structure of coloniality, which is an invisible underlying cause of the oppression of the ‘other’.

The article is premised on the specific concepts of coloniality of power and coloniality of being. The proponents of decolonial theory, among others, are Grosfougel (2007, 2011), Quijano (2000, 2007), Mignolo (2000, 2007, 2011), Maldonado-Torres (2007) and prominent scholars such as Ndlovu-Gatsheni (2001, 2013). Among other things, these scholars have the common agenda of exposing the ills of coloniality and Eurocentrism. Mignolo (2007:56) describes the decolonial theory as ‘an-other thought that seeks to inaugurate, “an-other logic”, “an-other language” and “an-other thinking” that has the potential to liberate’. In essence, decolonial theory is ‘an-another’ theory that comes from a different angle from CDS. It aims to bring awareness, liberation and agency for the oppressed social groups, such as people with disabilities, who are ‘othered’. Decolonial theory does not specifically explain disability issues, rather it explains issues of oppression of the ‘other’ which also include people with disabilities. Barton (2001) argues that we urgently need a political analysis and a theory of political action which is inspired by transformative change. Furthermore, Rose (2004) explains that decolonial theory does not only oppose coloniality, but it also proposes ways of overcoming oppression and prejudice. Thus, decolonial theory befits Barton’s (2001) and Rose’s (2004) descriptions of the social theory required in disability to change the oppression of persons with disabilities. It is not only radical against oppression but also offers a method of overcoming it. As many social theories have failed to bring change for persons with disabilities in terms of oppression (Oliver 1996), I therefore bring the decolonial perspective to understand obstacles for students with disabilities, in entry into professional degrees, and the underlying invisible causes by exposing the hidden structure of coloniality.

### Issues of coloniality

Decolonial theory seeks to create an understanding of oppression through understanding coloniality and its effects. It is an important premise when focus is on Africa in general, and in South Africa specifically, because humanity within this context has experienced flagrant colonialism and consequent oppression by colonisers. This particular theory helps to understand that although people in Africa live as ex-colonised people, they still live and breathe coloniality as global modern subjects (Maldonado-Torres 2007; Ndlovu-Gatsheni 2013). Quijano (2000:342) expresses a sentiment that ‘coloniality operates on every level, every arena and dimension of everyday human social existence’. According to Maldonado-Torres (2007), coloniality refers to:

… long standing patterns of power that emerged as a result of colonialism and have survived it. It defines culture, labour, intersubjectivity, relations and knowledge production. It is maintained in books, in criteria for academic performance, in cultural patterns, in common sense, in self-images of people, in aspiration of self and in so many aspects of our modern experience. In a way, modern subjects breathe coloniality all the time and every day. (p. 243)

As Quijano (2000) argues, it needs not to be overemphasised that some individuals in the context of Africa still live under coloniality. Although democracy has been achieved, some people are still subjected to coloniality, particularly in the South African context. Former disadvantaged social groups, like people with disabilities, have experienced gross oppression through the system of apartheid. This is why the present government wants to empower former disadvantaged social groups to access higher learning in general and professional degrees in particular. As such, it is therefore important to use decolonial theory to understand why students with disabilities are still hindered from entry into professional degrees. There are specific concepts of decolonial theory, which are important in terms of understanding access in this article. The theory has four constructs: coloniality of power, being, knowledge and nature (Dastile & Ndlovu-Gatsheni 2013). All the four constructs are important because they unveil how the structure of coloniality oppresses the other within a specific criterion. However, for the purposes of focus and scope of this particular article, the last two have been excluded and only the first two have been used to underpin this article. The first two specifically illuminate obstacles at entry for students with disabilities and the invisible underlying causes (RB, 3).

### Coloniality of power and zones of location

Coloniality of power proposes that despite freedom from colonialism in African countries, there are still networks of relations of exploitation, domination, control of labour, nature and its productive resources, knowledge and authority by the dominant powers (Quijano 2007). The existing colonial matrix of power affects all dimensions of social existence, ranging from sexuality, authority, politics, economy, subjectivity, language and race (Quijano 2000). This is why the South continues to be dominated by Western influences, and why oppression still continues for other social groups, despite democracy and proposed transformation.

Further to coloniality of power is the issue of social location of individuals within coloniality. It is explained in terms of zones, in which humanity is placed through what Santos (2007) terms the Western ‘abyssal thinking’. As Santos explains, this is a way of thinking by the West, which considers the social reality as divided into two realms. On ‘this side of the line’ is the zone of being, which is the metropolitan zone occupied by the West. The zone on the ‘other side of the line’ is the colonial zone, referred to as the zone of non-being, occupied by the ‘other’ (Santos 2007:45–46). The abyssal line is invisible but divides the two zones into differential power relations. Grosfougel (2011) explains that what is found in the two zones in terms of human race are two groups. In the zone of being, there are superior beings who are the ‘I’. The oppressors are found there. In the zone of non-being, there is the inferior being who is the ‘other’, the oppressed social group who exists as inferior beings. The humanity of the ‘other’ is denied. Knowledge and theories produced in the zone of being are claimed to be legitimate and universal. Dominant universities are located in this zone. In the zone of non-being, no credible and legitimate knowledge is produced; theories from this zone are discredited, including critical thinkers, critical theorists and critical scholars. In the zone of being, equality and freedom are considered rights, while conflicts are mediated through treaties, negotiation and law. In the zone of non-being, conflict and human relations are mediated and resolved through violence (Ndlovu-Gatsheni 2013). Grosfougel (2011) clarifies that the zones are the West, the zone of being, and the South, the zone of non-being.

The two zones should not however be understood in neatly formed, permanent categories. A zone of being can be created in the South through Western influence and education in general, and it produces the dominant society. Such people are socially located in the zone of non-being but epistemically located with the West. Grosfougel (2011) explains that they reproduce coloniality through confining themselves to a particular ethnic group in the zone of non-being, while they think and act like the oppressor in the zone of being. I argue that persons with disabilities could also be influenced by such positionality and location. By virtue of people with disabilities having the potential to oppress, they might be understood as oppressors rather than the oppressed. The concept of zoning, its complexities and dynamics are thus important to help not to over-generalise the obstacles for students with disabilities at entry. All students with disabilities might confront obstacles, but with diverse experiences of such obstacles because of the students’ epistemic location in terms of zones.

### Coloniality of being

The concept of coloniality of being derives from oppression of the way of being. It results from the categorisation of humanity into different social groups which are then labelled (Dastile & Ndlovu-Gatsheni 2013; Grosfougel 2007, 2011; Maldonado-Torres 2007; Mignolo 2007; Ndlovu-Gatsheni 2001, 2013; Quijano 2000, 2007). This categorisation is based on ideas of ‘normalcy’ and a binary view of abnormality and normality. This oppressive way in which humanity is categorised has resulted in the social construction of disability because persons with disabilities deviate from the standard of normalcy used in the process (Reddy 2011). They have been categorised and labelled as ‘the disabled’ because their bodies and minds deviate from the ‘normal’ body. The normative body is being used as a yardstick by the dominant powers responsible for social ordering of society, to name and discriminate people. The concept of ‘coloniality of being’ therefore brings an understanding to the issue of disability as a socially constructed phenomenon. It is in this way that although decolonial theory does not specifically explain the issues of disability, it brings to the fore the invisible underlying causes of oppression of persons who are ‘othered’. The theory of coloniality of being therefore situates the construction of disability in a wider theoretical account of othering. In the process of categorisation and producing normative standards, difference, multiplicity and diversity are denied (Ndlovu-Gatsheni 2012). The global population is thus ordered and differentiated by the dominant society into bipolar binaries of ‘inferior and superior, irrational and rational, primitive, civilised, traditional and modern’ (Quijano 2000:343). Thus, disability is a social construct produced through coloniality of being. However, the decolonial scholars (Grosfougel 2007, 2011; Maldonado-Torres 2007; Mignolo 2007; Quijano 2000, 2007) argue that all people are human. Differences in bodies, minds, race, gender, ethnicity and sexual orientation are all diversity in human beings, which should be celebrated and not denied. From the argument of decolonial scholars, it implies that ‘the disabled’ do not exist, neither do ‘the normal’. However, that the concept of disability has been constructed by society, and that people with disabilities socially exist, cannot be ignored. People with disabilities find themselves in the lower hierarchy of categorisation and susceptible to oppression; hence, there is the need to examine the obstacles they confront in terms of entering specific professional degrees in higher learning in South Africa. Decolonial theory would therefore not only promote the understanding of specific obstacles to students with disabilities at entry into higher learning, but also the obscured underlying causes of such obstacles. The theory is thus offered to address the social problem of exclusion or denial of access to students with disabilities.

### Problem and rationale of the study

The problem that was studied is that students with disabilities encounter obstacles to access. This fact remains despite the agenda of transformation and inclusion of diverse students in education in higher learning in a democratic South Africa. As such, prospective students with disabilities have been, and still continue to be, excluded from entering professional degree programmes. The obstacles that are seen at surface level may not be the real obstacles, or the only obstacles to entry for students with disabilities. This article proposes that the invisible, deep-seated results of coloniality can be viewed as contributory to the obstacles at entry for students with disabilities. The rationale of the study was, thus, to use decolonial theory to illuminate and expose the actual obstacles, which can consequently influence an effective intervention. Without such an exposure, oppression could be perpetuated **(**RB, 4).

## Research design and methodology

A qualitative research design was used in the study. This particular research paradigm was chosen to enable dialogue and development of partnership with research participants (Mertens 2009), which is important for collecting rich data. Qualitative research is philosophically hinged on the methodological assumptions of the transformative paradigm (Guba & Lincoln 1994), which allows the participants’ version of reality to emerge (Mertens 2010). The social contexts of participants, as defined by their schooling backgrounds, gender, race, socio-economic class, disability category and age, were taken into account, as these could influence how participants constructed reality. The voice of students with disabilities was privileged (Hosking 2008) to counteract the differential access to power in which the powerful’s version of reality is privileged (Mertens 2007). Privileging the voices of students with disabilities also allowed for the silenced voices to emerge. Participants were given an opportunity to state how they wanted entry to be improved. The opportunity to express their opinions in terms of improvement was important because their voices could be listened to, and heard, by stakeholders with authority when this research will be disseminated. This will be important because students with disabilities have the lived experiences of disability and its reality. The ethical and cultural values of the participants were respected (Mertens 2012), and to develop mutual trust and cooperation expected between the two parties, the researcher spent time in socialising with the participants before conducting the interviews. Two meetings were scheduled with each student before data collection commenced. One meeting was conducted at each student’s residence and the second at another agreed location. During these preliminary meetings, informal conversations were struck with participants to allow for a rapport to develop.

### Participants

Twelve students with disabilities and seven disability unit (DU) staff members (*n* = 19) participated in the study. Eight students were in their final year of study at undergraduate level and four were postgraduate students. Three students had hearing impairments, four had vision loss and five had physical disabilities and were using wheelchairs.

Sampling of participants was purposive. The particular students were selected because they had a lived experience of entering specific professional degrees at the institution. There were three members of the DU who were not disabled and four members with disabilities. The DU staff members were selected for their involvement and experiences with entry into specific programmes for students with disabilities. A letter of invitation was sent to DU members and those who volunteered to participate in the study responded. Students with disabilities were recruited through snowballing technique. Access to the first student was gained through a DU member who introduced the researcher to her. The researcher explained the study to the student and consent for participation was obtained. The student then referred the researcher to other students who could be interested to participate and provided their names. The researcher then introduced herself and the study to each one of them. Participants of different races, gender, ages and schooling background were included in the sample, resulting in maximum variation being attained.

### Data collection

Qualitative data were collected through in-depth individual interviews by the researcher. The interviews were unstructured, which Gubrium and Holstein (2002) argue are more flexibility, and were conducted on a one to one basis. Although Frith (2000) argues that participants talk more freely on sensitive issues in focus group interviews, for students with various disabilities to be interviewed in a group was viewed less likely to yield rich data. They would, as Kitzinger (1994) observed, question each other and try to persuade each other to one’s point of view. A semi-structured interview guide, developed from the research questions of the study, was used. The interviews were conducted at one formerly advantaged institution of higher education, with a DU anecdotally regarded as one of the best units in the country associated with the institution.

Interviews were conducted during 2015. Five students with disabilities and four DU staff members were first language English speakers, and seven students and three staff members were second language English speakers. All participants were, however, interviewed in English because it was a common language understood by the interviewer and all participants. There were no communication barriers between the interviewer and participants with hearing impairments because all of them used oral communication. Each interview was conducted at a place and time convenient to the individual participant. Interview data were audio-recorded with the permission of the participants and transcribed and the verbatim transcripts were returned to participants for verification.

Data were analysed thematically (Byrne 2001; Creswell 2008; Leedy 1997), and at different levels, by the researcher. At each level, similar responses were aggregated and collapsed into themes. Similar views and contradicting views were grouped together, and trends were analysed. The first stage of analysis involved the researcher analysing data for minor themes. They were grouped together and abstracted to major themes (Braun & Clarke 2006; Miles & Huberman 1994). Access and alienation, intersectionality, equality and inequality, and tensions of disability and impairment were abstracted as major themes.

Cross-checking of data, which Ndhlovu (2014) refers to as ‘constant comparative analysis’, was extensively used during the study. It helped to identify contradictions and consistencies in the data. Responses from students with disabilities were constantly compared with those of the DU staff members. As students with disabilities are not a homogeneous social group, their responses from the context of different schooling backgrounds, economic class, race, gender and disability categories were also compared. It was important for understanding intersectionality among students with disabilities as a factor in shaping their experience of gaining entry into the institution (RB, 5). Peer reviews and member checks with colleagues in the field were also used to validate the analyses. Triangulation (Carter et al. 2014) was used to validate data from different sources. Data from the DU staff members were triangulated against that from students with disabilities. Data from students with disabilities from different schooling backgrounds were also triangulated.

### Reflexivity

As the researcher had more control of the study and was the one who gathered and analysed the data, there could have been researcher biases. I identify strongly with students with disabilities because of my personal experiences of exclusion. Thus, I had a vested emotional interest in the study because of shared experience of segregation and being discriminated. There could be a possibility of my being more attuned to experiences of exclusion than to those of inclusion in the process of collecting and analysing the data. I, however, made an effort to be neutral by recording all interviews using a digital recorder. Use of mechanical methods reduces researcher bias (Breakwell, Hammond & Fife-Shaw 1995) because recorded data can be transcribed verbatim. Recording the interviews reduced the insider effects. However, subjectivity is also acknowledged as a researcher effect. Perfect neutrality and objectivity are simply not possible, given the human element of such a study. Knowledge production cannot be totally value-free, as it cannot be independent of the researcher producing it (Berger 2015). Thus, there could be researcher effects in the results of the study as subjectivity cannot be totally avoided.

### Ethical considerations

Ethical considerations were strictly followed to limit the vulnerability of students with disabilities, as much as possible. Permission to conduct the study was obtained from the institution of higher learning, which was also the site of research, and ethics clearance was granted by the ethics committee (clearance number 2013CE106D). Informed consent was sought from all the participants and the nature, purpose and aim of the study were explained to them. Participants were made aware that their participation was voluntary and that they had the right to withdraw from the study should they feel unwilling to continue.

## Findings of the study

Contradictory views were expressed by the participants on ease of entry of students into the specific professional degrees at the institution. The contradictions were revealed between students with disabilities having access to entry into professional degrees and those having experiences of alienation. Experiences of both equality and inequality were shared, and the influence of intersectionality became apparent as a theme in the findings. The final theme depicts the tension that was found between the presence of impairment and the experience of disability, as these impacted the entry of students with disabilities into professional degrees.

### Access and alienation at entry

Students with disabilities had contradictory views on their entry into specific professional degrees. All 12 students with disabilities across the three programmes agreed with DU members that academic merit was the primary criterion for access. They stated that all students had to meet the entry requirements in order to enter. They also shared the same view as DU staff members that policy afforded them an opportunity equal to that of students without disabilities. One of them said:

‘I did not experience any problems myself to enter into Education because I had the entry requirements they needed. I did not struggle to get in. I had all the subjects and the points and so it was easy for me to enter.’ (Student of Education: 4, female, 19 years old)

Although students with disabilities had initially said that access was possible with the required marks and subjects, an experience of alienation was reflected in their reports about the challenges in their school careers, which they feel were not encountered by students without disabilities, and culminated in barriers or challenges to entry at the level of higher learning. Eight out of the 12 students stated that special schools limited their likelihood of gaining entry to the higher degree they wanted to pursue because some schools did not offer subjects specifically required for Medicine. Those who were studying Medicine and Law stated that had they not gone to mainstream schools, they would not have entered those programmes. One of them said:

‘Special schools and disadvantaged schools have no prospect of bringing disabled students who qualify to do Medicine at this university. So the barrier can be the school that you come from.’ (Student of Medicine: 1, Male, 26 years old)

Contrary to their initial statements that they also had equal opportunity to enter a professional degree, students with disabilities stated that they needed special concessions so that they also had equal opportunities at entry. One of them stated:

‘I want them to make special consideration in entry requirements and admissions because you can’t pretend you don’t have a disability.’ (Student of Education: 5, male, 23 years old)

Disability unit members said that the specific professional degrees were accessible to students with disabilities at entry because the policy did not allow discrimination on the grounds of disability. They stated that if students with disabilities provided prerequisite entry qualifications, they had the same opportunity of entry as that of any other student. One staff member commented:

‘Since I have been here for 16 years to be precise, I have never had an experience of a student who is discriminated against because he has a disability.’ (Disability Unit member: 4, male, 46 years old)

While they had said access was equal, DU members also agreed that entry into professional degrees, and Medicine specifically, was difficult for students with disabilities at the institution, as captured by the following statement:

‘From my experience, students with disabilities are the ones who struggle very much to get into the professional degrees at this institution. The subjects they have do not fit the entry requirements for professional degrees as Medicine.’ (Disability Unit member: 2, male, 24 years old)

Another member of the DU presented a perception of alienation at entry:

‘Students with disabilities have problems entering professional degrees because they might not be doing the required subjects in their Matric. Even teachers have low expectation that those students can do challenging subjects.’ (Disability Unit staff member: 3, female, 29years)

### Intersectionality

Because of issues of class and privilege, there were students with disabilities who did not encounter any obstacles in entering professional programmes of their choice at the institution. Despite their disabilities, they attended mainstream schools and obtained the prerequisite marks and subjects, which enabled their entry into the specific programme they wanted. A medical student stated:

‘I had always wanted to be a doctor and I went to a normal school. I studied maths, biology and chemistry. I did this subject integration because Medicine is the only degree I ever wanted.’ (Student of Medicine: 1, male, 26 years old)

Intersectionality also manifested in different special schools attended by students with disabilities. The participants stated that it was specifically those from special schools for the Deaf who did not meet the entry requirements in terms of marks and subjects for specific professional programmes. They said that other special schools offered the same subjects as mainstream schools, and hence afforded opportunity for students with disabilities to enter their degree programme of choice, just like any other student. Three out of the 12 students said they had not encountered any obstacle in entering professional programmes of their choice, although they had attended special schools. One of them stated:

‘You find that in many Special schools, they are not doing Maths. At our school, which is a special school, the one I matriculated from, I did every subject that is offered in the mainstream.’ (Student of Education: 6, female, 21 years old)

The above statements show that experiences of students with disabilities cannot be generalised as there are factors that intersect with the presence of a disability and influence different students’ experiences differently. Thus, we find some students with disabilities enter the programme of their choice, despite their disabilities, because of high socio-economic class, privileged position and the type of special schools the students attended,.

### Equality and inequality at entry

Students with disabilities reported that they were afforded equal access into the professional degrees for which they qualified. This was shown in the below statement:‘

The university values your personal ability in terms of intellectual self. Just the necessary points, I did not have any problems because I had the points they wanted.’ (Student of Education: 1, female, 23 years old)

The perceptions of students with disabilities were that there was equality because they could also meet the requirements like all other students, as expressed by one participant:

‘I was not treated like a disabled student. I met the academic requirements so that I could get into Law. It would be unfair for me to enter Law because I am on wheelchair. There was no special consideration for me to come in.’ (Student of Law: 3, female, 26 years old)

The above statement suggests that the student thinks there is equality in the same entry requirements because he or she is also capable. While it could be seen that way, students with disabilities encounter the obstacle of inequality because they presumably have disadvantages stemming from their school careers, which other students do not have. Students with disabilities from some special schools, for example, are already denied entry into Medicine and Law specifically, because of high entry points and the requisite subjects of sciences. This is inequality because the playground is uneven for students with disabilities. The inequality is invisible but it prevents access at the point of entry. An observation was made in this regard:

‘You find that in many special schools they don’t do maths and science subjects. Definitely you would not enter Medicine. You end up doing the degree you don’t want.’ (Student of Law: 2, male, 20 years old)

This statement confirms an issue of inequality that is invisible because students with disabilities are limited in terms of entry to the professional degrees of Medicine. Thus, while at surface level, all students seem included and there is equality in terms of entry requirements and policy, students with disabilities are not fully included.

From the DU staff members’ perspective, there seemed to be equal access to the specific degrees because entry requirements were the same for all students, those with and without disabilities. There was no special consideration for students with disabilities.

### The tension of impairment and disability

The perception at the institution was that specific impairments limit entry into particular programmes. For example, the perception of the DU staff and students with disabilities was that an individual with visual and hearing impairments could not enter the programme of Medicine. A student with a hearing impairment in Medicine stated:

‘How am I supposed to use a stethoscope, how am I supposed to interview patients, it kept ringing in my mind, a doctor has to hear, hear, hear, I can’t be a doctor.’ (Student of Medicine: 2, male, 26 years old)

A student with a visual impairment in the programme also stated:

‘I cannot operate on a patient with this vision. I cannot do procedures that really need good sight.’ (Student of Medicine: 1, male, 24 years old)

The student had started his medical degree at a university in Latin America, and he stated that had he started at the institution under study, he might not have entered Medicine. The utterances of the two students with hearing and vision loss suggest that they were convinced that their impairments limited entry into the programme. Furthermore, dyslexia and communication disorders were viewed as impairments that hindered entry into an Education degree. They stated that a student with communication, reading and writing limitations might not be able to teach those same skills to learners in schools and might not be able to write reports, letters to parents and speak clearly to learners and parents.

Students with disabilities also perceived a speech disorder as an obstacle to entry into Law because the profession required someone who was articulate and fluent to represent clients well. They also viewed physical disability as limiting at entry because of the dress code that was required in Law. One of them said:

‘Law is appearance driven (RB, 6), you have to dress in a certain manner, you can’t come with a leg brace over your jeans and you can’t afford not to wear suits and stuff.’ (Student of Law: 3, male, 21 years old)

## Discussion

This study has found that although efforts are being made for students with disabilities to enter higher education, there are inequitable structures and practices that limit their entry to professional degrees at the institution under study. When illuminated by decolonial theory, the opportunity of access that students with disabilities have through policy can be explained in terms of efforts of transformation and inclusion. Dastile and Ndlovu-Gatsheni (2013) argue that the importation of human rights and democracy from the West has resulted in a shift towards transformation and inclusion in the neo-colonial present. As South Africa has attained independence and democracy, it could be argued that the country is making an effort through inclusive legislation and policies to transform its higher learning institutions to include diverse students. To redress the inequalities of the past, the institution abides by the policy of non-discrimination and inclusion (*Equality and Prevention of Unfair Discrimination Act* 2000), and Education White Paper 6: Special Needs Education (EWP6) (DOE 2001). Thus, non-discrimination and equal access in terms of academic merit seem to promote equal access to professional degrees for all students, including those with disabilities. Evidence from this study reflects that students with disabilities think that they are afforded equal access into the professional degrees that they qualify for. Dastile and Ndlovu-Gatsheni (2013), however, argue that inclusion of all is illusionary at present.

Alienation of students with disabilities who come from special schools can be understood in light of location in different zones, as explained by Santos (2007) and Grosfougel (2011). Special schools are located in the zone of non-being, while the institution of higher learning, by virtue of being previously advantaged, is in the zone of being. Thus, students with disabilities are alienated by the invisible ‘abyssal line’ (Santos 2007), which distinguishes special schools, designed for the other, from a dominant university (an institution on the zone of being and understood as elite). It is nevertheless expected that students with disabilities achieve the same entry requirements as students without disabilities to enter Medicine at the institution, while their schooling background is different from students without disabilities. Ndlovu (2015) argues that coloniality thrives on alienating the other. Dominant universities are also used as power structures to sustain coloniality and to oppress the other (Dastile & Ndlovu-Gatsheni 2013). The alienation of students with disabilities could thus be explained in light of coloniality sustaining itself. I argue that, while at surface levels the same entry level requirements seem to provide equal opportunity to all students, at deeper unseen levels the invisible underlying cause of alienation of students with disabilities is the zone of non-being, in which they are located.

Although Howell (2005, 2006) argues that there has been improvement in schooling to promote access to higher learning by students with disabilities, I argue that entry into the specific professional programme at the particular institution is still limited by virtue of alienation caused by limitations in the special schooling system. As revealed in the data, some special schools do not offer subjects that are prerequisites to enter specific professional degrees in higher learning. From the American context, Trow (2000) argues that alienation is experienced because of incomplete transformation from systems of dominant universities to systems of mass higher education that provide universal access. I argue that access into higher learning by diverse students has indeed increased in South Africa; however, students with disabilities in particular are still limited in entering specific professional degrees.

Intersectionality had a different influence on the entry of students with disabilities into the three programmes. When the influence of intersectionality in obstacles confronted by students at entry is illuminated by decolonial theory, it could be understood in terms of fluidity of zones of location (Grosfougel 2011). Not all students with disabilities are rigidly confined to the zone of non-being. There are some students who by virtue of socio-economic class, race and impairment category and its level of severity are socially located in the zone of being. Such students may not be hindered in terms of access into specific professional degrees at the institution, and may be privileged rather than oppressed. As Mertens (2009) states, intersectionality can also privilege and does not always yield double oppression. Access and alienation are therefore experienced differently by students with different disabilities and thus it should not be generalised to all students with disabilities that they are alienated at entry.

Issues of equality and inequality are revealed at the point of entry, but inequality seems to be invisible to the participants. When decolonial theory is used as a lens to illuminate the invisible inequality at entry, it could be explained in terms of coloniality of power. As already highlighted, the dominant ethos used at the universities sustain the oppressive structure of coloniality, and to continue to oppress ‘the other’ (Grosfougel 2011). Powerful programmes and powerful knowledge are being offered to the powerful. At surface level, the exclusion to enter Medicine by students with disabilities from special schools and disadvantaged mainstream schools could be seen as resulting from not having the required subjects and marks. However, beneath the surface level, it can be seen as a way of keeping the powerless from powerful knowledge. Those who have access to powerful knowledge are the powerful, and not the oppressed in the zone of non-being. Dominant universities have the obligation to maintain this status quo. While the same entry requirements and admission procedures for all students may seemingly afford equal opportunity to all, indeed it does not. It is an issue of maintaining power, that power remains with the powerful and the oppressed remain powerless. It is therefore important that the issues of obstacles to the access of students with disabilities into professional degrees are analysed deeply.

Another obstacle at entry for students with disabilities is the tension of impairment and disabilities. The two are taken together as a social construct by critical disability scholars, such as Tremain (2005), while they are actually different. Decolonial theory helps us understand the tension between impairment and disability in light of the organisation of society using ‘normalcy’ as the standard (Quijano 2000). Physical structures, practices and the general order of society are organised for ‘normal’ people, hence excluding those with different categories of impairment. For example, the perception of students with disabilities is that speech disorder is an obstacle to entry into Law because the profession requires someone who is articulate and fluent to represent clients well. By virtue of being excluded by inaccessible structures, some students with disabilities think their entry is limited because of their impairments, not realising that it is society that excludes them. For example, the dress code which excludes those with physical disabilities is conceived as an issue of impairment by a Law student. Maldonado-Torres (2007) explains that there are oppressed social groups who are and have been living under oppressive powers and have accepted them as realities of modernity. The participants’ view that specific impairment hinders entry could be seen in light of internalised oppression (Hall 1990; Mason 1990; Reeves 2014; Thomas 2007). I argue that there is a reproduction of the understanding that impairments result in disability and, consequently, disability is seen as inability. Students with hearing and visual impairments studying Medicine perceived that fulfilling the requirements of the profession would be impossible for them because of their impairments. While at surface level it makes sense, a question could be asked as follows: who said doctors should be hearing or seeing people because humanity is diverse, plural and different? (Ndlovu-Gatsheni 2001; Quijano 2000). It must be understood that impairment and disability are not the same. From the social model of disability perspective, it is society that disables and limits, and not impairments.

The tension between impairment and disability was evident across the three programmes when students described how exclusionary the designs and demands of the specific programme were for them. However, they continued to emphasise their own specific impairments as the restrictors to entry in particular programmes. Crow (1996) in agreement with Shakespeare (2010) argues that when an impairment, rather than a disabling condition, is emphasised as limitation, it is an obstacle in itself. The focus of the responsible authorities will shift from transforming an exclusive context of learning to transforming the students to suit the context. Oliver (1990) views this perspective as individually oriented. I concur with Shakespeare and Oliver by arguing that it is not the impairments of the students but the structure of the specific professions and the designs of the programmes that pose obstacles that limit students with particular impairments to enter specific programmes. The view of specific impairments as a hindrance to entry into specific professional degrees could be seen as a reproduction of individualised understanding of self (Devlin & Potheir 2006) that has been internalised. Students with disabilities could therefore unconsciously exclude themselves from entering the specific professional degrees at the institution by pre-judging themselves before they are judged by others. The tension of impairment and disability is reflected in the students because they appear to conceptualise disability and impairment as the same.

## Way-forward: A step towards intervention

The real obstacles, which include the effects of coloniality as categorisation and hierarchisation of people, denial of difference and use of normative standards for all diversity, are much broader and deeper than what could be seen by ‘a naked eye!’ Specific interventions are therefore suggested to improve entry of students with disabilities into professional degrees in higher learning broadly. With specific reference to Botswana, Habulezi and Phasha (2012) suggest adaptations to teaching approaches as an intervention at school level to counter the alienation and inequality confronted by students with disabilities. In the South African context, I suggest levelling the playing field at the point of entry for people with disabilities should rather entail reasonable accommodation measures, which are already described in policy but not yet implemented adequately and effectively. The implementation of equity of access stipulated in the policy of transformation in higher learning (DOE 1997) is one measure that could increase the access of students with disabilities at entry (RB, 7).

There is also a need to improve special schools. It has been confirmed from the data that students with disabilities from such schools are denied access to higher education in general and professional degrees specifically. McKinney and Swartz (2016) revealed that during the apartheid era, special schools were also divided according to race, with white schools receiving better education. It suggests that special schools differ in the way students with disabilities are educated. An attempt has been made to improve formerly black special schools by the democratic government through the Inclusive Education policy. It is recommended, as McKinney and Swartz (2016) extrapolated, that the principles of Inclusive Education (EWP6, 2001) should be effectively implemented for the improvement of special schools that are disadvantaged (RA, 1).

There is also a need to work together because the war of coloniality is far too great to be won single-handedly by a single social group. Oliver and Barnes (2012: 176) also argue:

Oppression of disabled people will only end when the oppression of all is overcome and that will happen with major structural, economic, political and cultural transformation as well as resistance. (p. 176)

Oliver and Barnes’s proposition of a transformation, in which students with disabilities could access professional degrees, is broad and might take very long to accomplish, taking into account the resistance that could also be encountered in the process. I suggest that dismantling coloniality should start at the institution, that there is a total institutional transformation in terms of structures, culture and practices, in which all diverse students are included at entry. It could be an intervention which could see even students with disabilities having a wider access to the three programmes specifically, and any professional degree of choice broadly, in the South African higher education sector. Furthermore, intersectionality should not be glossed over because obstacles at entry into professional degrees are not the same for all students with disabilities. Thus, when stakeholders at institutions of higher learning and students with disabilities become aware and conscious of coloniality, they can begin to reveal its ills and spearhead resistance and transformation **(**RB, 8).^1^

## Conclusion

If the invisible underlying causes of obstacles at entry are not visible to students with disabilities themselves and those involved in access issues, they might continue to be excluded from entering the specific professional degrees at the institution. The inequitable practices and structures can only be dismantled when there is awareness and consciousness that the deep-seated cause of the obstacles confronted at entry has to do with coloniality. Consequently, ‘treating the underlying cause’ would be the coming together of all oppressed social groups and non-disabled persons who are also fighting oppression to engage coloniality. Intervention at national level (RB, 9) would be a drive to a total overhaul of the tertiary education system and complete institutional transformation to include all diverse students to access professional degrees, learn and graduate. Graduates with disabilities could thus also enter professional employment and contribute to professional skills for a global, diverse market as expected in a democratic country (RB, 10).

## Footnotes

1 Equity of access is addressed in a number of clauses in policy. It states that the goal of transformation in higher education is to give fair chances of access to all students who have the potential. Furthermore, it states that there should be eradication of all forms of unfair discrimination (DOE 1997). It implies therefore that all diverse students including those with disabilities cannot be denied access into higher education generally. If there could be an effective implementation of this clause, students with disabilities could have an equal opportunity of access into professional degrees in higher education.
